# Nanoparticles as Drug Delivery Systems for the Targeted Treatment of Atherosclerosis

**DOI:** 10.3390/molecules29122873

**Published:** 2024-06-17

**Authors:** Alexander Shao-Rong Pang, Tarini Dinesh, Natalie Yan-Lin Pang, Vishalli Dinesh, Kimberley Yun-Lin Pang, Cai Ling Yong, Shawn Jia Jun Lee, George W. Yip, Boon Huat Bay, Dinesh Kumar Srinivasan

**Affiliations:** 1 Yong Loo Lin School of Medicine, National University of Singapore, Singapore 117597, Singapore; alexandersrp@u.nus.edu (A.S.-R.P.); natalieylpang@gmail.com (N.Y.-L.P.); cailing2002@gmail.com (C.L.Y.); 2 Department of Medicine, Government Kilpauk Medical College, Chennai 600010, Tamilnadu, India; tarinidinesh@gmail.com; 3 Department of Pathology, Dhanalakshmi Srinivasan Medical College Hospital, Perambalur 621113, Tamilnadu, India; vishallidinesh@gmail.com; 4 Division of Medicine, South Australia Health, Northern Adelaide Local Health Network, Adelaide, SA 5112, Australia; pang.kimmy@gmail.com (K.Y.-L.P.); shawnleejiajun@hotmail.com (S.J.J.L.); 5 Department of Anatomy, Yong Loo Lin School of Medicine, National University of Singapore, Singapore 117594, Singapore; antyipg@nus.edu.sg (G.W.Y.); antbaybh@nus.edu.sg (B.H.B.)

**Keywords:** atherosclerosis, nanoparticles, nanotechnology, targeted therapy, drug delivery systems

## Abstract

Atherosclerosis continues to be a leading cause of morbidity and mortality globally. The precise evaluation of the extent of an atherosclerotic plaque is essential for forecasting its likelihood of causing health concerns and tracking treatment outcomes. When compared to conventional methods used, nanoparticles offer clear benefits and excellent development opportunities for the detection and characterisation of susceptible atherosclerotic plaques. In this review, we analyse the recent advancements of nanoparticles as theranostics in the management of atherosclerosis, with an emphasis on applications in drug delivery. Furthermore, the main issues that must be resolved in order to advance clinical utility and future developments of NP research are discussed. It is anticipated that medical NPs will develop into complex and advanced next-generation nanobotics that can carry out a variety of functions in the bloodstream.

## 1. Introduction

### 1.1. Atherosclerosis

**Figure 1:**
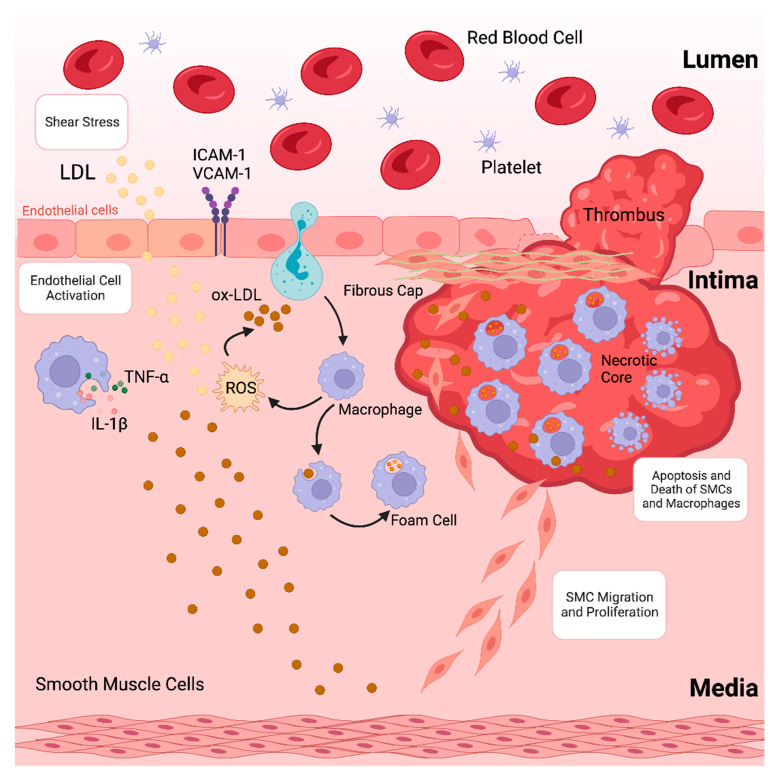
Formation of an atherosclerotic plaque.

Atherosclerosis, characterised by the accumulation of plaques inside the arterial wall and accompanied by dysregulated lipid metabolism, is among the leading causes of morbidity and death globally [1]. The development of atherosclerotic plaques, which causes blood arteries to narrow and impede blood flow, is often the underlying cause of coronary artery disease (CAD) [2,3]. Atherosclerosis is a chronic inflammatory disease caused by the accumulation of lipoproteins, containing plasma apolipoprotein B (apoB) in specific regions of the arterial tree [4]. These lipoproteins contain cholesterol and oxidised phospholipids that activate endothelial cells (ECs), which then draw monocytes into the subendothelial space [5,6]. Monocytes in the arterial intima undergo differentiation into macrophages that promote inflammation, which in turn intensifies the inflammatory response locally [7]. Furthermore, lipoproteins in the intima are consumed by macrophages, resulting in lipid-laden foam cells that initiate atherosclerotic lesions. Increased macrophage apoptosis and ineffective apoptotic cell clearance are signs of an advanced stage of atherosclerotic lesions that develop if the proinflammatory state continues (as depicted in Figure 1) [8,9,10]. This deadly mixture encourages plaque necrosis, a crucial characteristic of vulnerab8-le plaques that can result in occlusive luminal thrombosis and its aftereffects, which include myocardial infarction (MI), stroke, and abrupt cardiac death [11,12].

The long-term adverse effects of atherosclerosis continue to be the principal cause of mortality in both developed and developing countries [13]. Diabetes, smoking, hypertension, hyper-lipidaemia, and a poor diet are several predisposing risk factors for atherosclerosis. Multi-factorial CAD risk factors include genomic, biological and behavioural factors [14]. CADs are often dubbed as the ‘silent killer’ because they are frequently clinically asymptomatic, and hence are missed or overlooked [15]. Theoretically, lowering apoB-containing lipoproteins to extremely low levels early in life could eliminate CAD. Unfortunately, due to low compliance and adverse effects that could arise from this approach in some individuals, this strategy is currently not practical for widespread use. Furthermore, a thorough assessment of the safety issues surrounding the use of cholesterol-lowering medications in children is still pending a full evaluation. To further lower the risk of CAD, new anti-inflammatory therapies are being developed. However, high toxicity, low target specificity, and decreased bioavailability of conventional anti-inflammatory drugs hinder these efforts. It is still difficult to determine which groups of people are susceptible to developing clinically dangerous vulnerable plaques [16].

### 1.2. Nanotheranostics

Nanotechnology is the study and manipulation of colloidal matter in the dimensional range of 1–100 nanometres (nm). Because of their size and surface functions, nanomaterials have a variety of properties [17]. Nanomedicine is the biomedical application of nanotechnology for diagnosis, treatment, and prevention of diseases at the cellular and molecular level through the use of precisely manufactured materials at this length scale to create novel diagnostic and therapeutic modalities [17,18].

**Figure 2:**
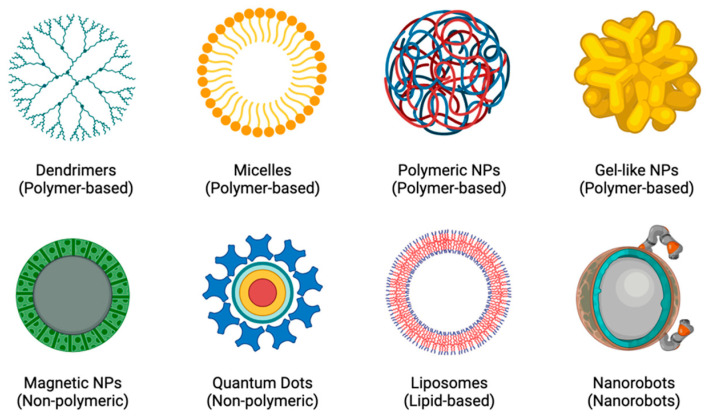
Examples of different types of NPs.

In order to create more individualised and personalised therapeutic procedures, nanotheranostics (a concept that combines both therapy and diagnostics) was created [19]. The rationale is based on the principle that diseases, such as CADs and cancers, are highly diverse and heterogeneous and that the majority of patients only respond well to current treatments when their conditions are in specific stages of progression. Developing a close link between diagnosis and treatment would lead to more individualised treatment plans for patients, which would greatly improve their chances of recovery [19,20]. The potential for molecular imaging and targeted therapy has increased with the rapid development of nanotheranostics and the availability of a wide range of novel agents, including polymer conjugations, dendrimers, micelles, liposomes, nanoemulsions, self-nanoemulsifying drug delivery systems (DDS), and metal and inorganic NPs [19,21], as shown in Figure 2. Because of their long circulating half-lives, binding selectivity, low toxicity profiles, ease of manufacturing, and potential for delivering complementary therapies, NPs are excellent tools for enhancing theranostic approaches to treating diseases [22,23].

Nanomedicine has filled the pipelines of the pharmaceutical industry with an entirely new set of therapeutic options. Therefore, some of the anticipated medical applications include DDS, ex vivo and in vitro diagnostics, nutraceuticals, and the development of better biocompatible nanomaterials [19]. 

### 1.3. Nanoparticles in the Detection and Treatment of Coronary Artery Diseases

Although patients have yet to be treated with NPs for atherosclerosis in the clinical setting, we believe that future clinical translation of nanomaterials for CADs will be accelerated by the increased understanding of atherosclerotic pathophysiology that has been acquired over the past few decades, along with the success of nanomedicines in cancer therapy. The advancements, challenges, and future directions in the rapidly evolving field of NPs will be discussed.

**Figure 3:**
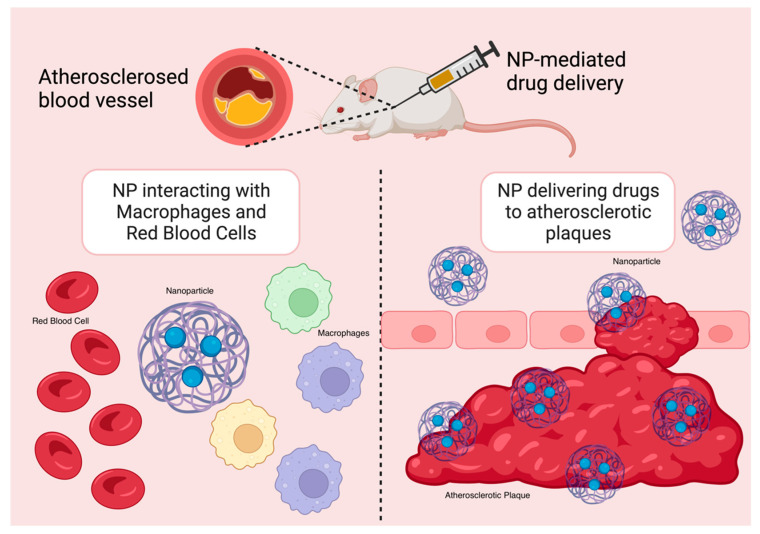
NP targeting atherosclerotic plaques in mouse models.

A wealth of research has demonstrated that NPs significantly enhance the chemical stability and pharmacokinetic profile of loaded therapeutics, including small-molecule pharmaceuticals, peptides, proteins, small interfering RNA (siRNA), and microRNA (miRNA). NPs are hence particularly useful tools in anti-inflammatory therapies in the treatment of CADs. By lowering off-target and systemic side effects in comparison to free drugs alone, nanotherapeutics may also help drugs overcome major translational obstacles [16,24]. The delivery of therapeutics and imaging agents to inflammatory macrophages in atherosclerotic plaques has been improved with the use of targeted NPs (Figure 3). Moreover, in mouse models, NP-mediated drug delivery strategies have demonstrated great promise in suppressing certain pathological processes, such as inflammation, that are linked to the advancement of atherosclerosis with a high degree of specificity [16].

Several studies and clinical trials have shown that using nanomedicine-enabled techniques to identify vulnerable atherosclerotic plaques and potentially vulnerable CAD patients holds enormous potential for the creation of efficient nanotheranostic strategies for atherosclerosis and CADs [25,26]. Designer NPs with targeting ligands have been developed for plaque and heart-targeted drug delivery to combat CADs. These nanocarriers transport drugs to a specific therapeutic spot without harming healthy tissue [15]. The theranostic applications of NPs in CADs could close the gap between experimental data and extensive clinical trials. Nanotheranostics in cardiology enable image-based therapeutic DDS, thus integrating medications with imaging [27]. The challenges of conventional systemic drug administration in CADs, which include poor bioavailability and absorption, instability, and undesirable adverse effects, could be overcome using nanotechnology-based techniques. In the repair and regeneration of vascular tissue, the use of DNA vectors, miRNAs, and stem cells has also been impeded by inefficient delivery [15].

Site-specific uses in magnetic resonance imaging (MRI) have been made with regard to vascular intervention. It has been claimed that magnetic NPs can detect and halt inflammatory processes in atherosclerotic plaques [28]. Natural compound-coated magnetic NPs are effectively used in the imaging of CADs. For example, arterial thrombus molecular imaging by MRI is performed using ultrasuperparamagnetic iron oxides (USPIOs) coated with fucoidan, a polysaccharide. As observed using an elastase-induced murine vascular injury model, this contrast agent is capable of detecting and visualising P-selectin, an adhesion molecule, as the molecular component of atherothrombotic vascular disease [29]. Additionally, gold nanorods have been employed in photodynamic therapy (PDT) to detect and attenuate macrophages [30,31]. 

Nanomaterials possess unique characteristics, such as high surface plasmon resonance (SPR), a high surface-to-volume ratio, tuneability, biocompatibility, and optical and magnetic properties, which contribute to their significant impact in disease diagnosis, and as localised therapeutics in CAD treatment [32]. The clinical challenges posed by CADs provide an opportunity for the application of nanotheranostics. Given that atherosclerosis is the primary cause of cardiovascular-related fatalities, significant efforts have been dedicated to the development of numerous nanotheranostic biomaterials for the treatment of CADs over the past few decades [33].

We offer a novel viewpoint on nanotheranostics for the treatment of atherosclerosis in this comprehensive review, outlining the contribution of liposomes, dendrimers, micelles, polymeric NPs, gel-like NPs, magnetic NPs, quantum dots, and nanorobots in the quest for improved nanotheranostic solutions to address the growing epidemic of CAD due to atherosclerosis. In particular, we explore how advancements in the diagnosis and treatment of atherosclerosis may be driven by the convergence of nano-technology and a better comprehension of the pro-atherosclerotic mechanisms mediated by macrophages in atherosclerotic plaques. 

## 2. Nanoparticles for Diagnosis and Treatment of Atherosclerosis

Over the past decade, there has been notable advancement in the design and development of innovative nanotheranostic agents aimed at diagnosing and treating atherosclerotic plaques. These agents are designed to operate on a shared platform, effectively combining both diagnostic and therapeutic capabilities. The positive outcomes observed in preclinical studies of atherosclerosis and the successful utilisation of nanomedicine-based strategies in human cancer treatment are promising indicators of their potential future application in diagnosing and treating patients with atherosclerosis [16].

In recent years, there has been significant interest in the potential of nanotheranostic platforms for the targeted image-guided treatment of CADs. Nanotechnology offers significant advantages in enhancing the pharmacokinetic characteristics, and chemical stability of enclosed diagnostic and therapeutic agents for the management of atherosclerosis [16]. These promising approaches involve the use of spatially designed nanosystems, which are regarded as highly effective tools for combatting atherosclerosis [32]. The four major concepts of atherosclerosis-based nanotheranostics encompass targeting, multi-functionality, high local drug concentration, and controlled drug release. The present treatment approaches for atherosclerosis concentrate on stabilising vulnerable plaques, which are characterised as plaques that have a high probability of rupturing and causing thrombosis [34]. The development of imaging agents based on NPs, which can precisely target inflammatory macrophages within atherosclerotic plaques, holds diagnostic promise for the non-invasive measurement of atherosclerosis plaque accumulation. It also enables assessment of the effectiveness of medical treatments and serves as a surrogate endpoint. Nanotheranostics that are designed for precise targeting and capable of influencing the functions of plaque macrophages, through the activation or inhibition of specific signalling pathways, have demonstrated significant potential in preclinical models. Taking such an approach could enhance therapeutic effectiveness while minimising off-target and systemic side effects [16].

New kinds of stents that serve as drug-eluting stents (DES) have been developed to lower the danger of restenosis and or in-stent thrombosis brought on by bare-metal stents (BMS). To inhibit the growth and proliferation of smooth muscle cells, a large variety of diverse medications, including anticancer and anti-inflammatory medicines, have been explored in combination with DES. An alternative strategy is to alter gene expression using plasmid DNA or RNA interference to create an imbalance in specific signalling molecules locally, thereby preventing some cells from proliferating while boosting growth of other cells [35]. Furthermore, Gundogan et al. (2014) investigated pH-responsive antioxidant administration for the treatment of atherosclerosis [36].

Recently, there has been a significant focus on United States Food and Drug Administration (US FDA)-approved NPs, such as poly lactic-co-glycolic acid (PLGA), hyaluronic acid (HLA), and liposomes, for the treatment of atherosclerosis. These nanomaterials have garnered considerable attention due to their ability to mimic the characteristics of high-density lipoproteins (HDLs), which are known for their anti-atherogenic properties [37]. Amine-functionalised HA-NPs that are labelled with either a fluorescent or radionuclide agent have demonstrated the effective promotion of macrophages’ efficient uptake and facilitated the elimination of atherosclerotic plaques. The presence of fluorescence agents, serving as contrast agents, offers promise for various bioimaging modalities [38]. Likewise, polymeric NPs and HDL-mimicking NPs have also become the focus of attention for investigating their potential in bioimaging and therapeutic approaches for the management of atherosclerosis [39].

### 2.1. Nanoparticle Drug Delivery Systems in Atherosclerosis

Many inflammatory disorders, including atherosclerosis, have been linked to the interleukin-1β (IL-1β) pathway [40,41]. Currently, only downstream targeting of IL-1β or the systemic neutralisation of IL-1β itself with scavenging antibodies such as canakinumab are used in the clinic [40]. An alternate approach that shows great promise is targeting IL-1β upstream by inactivating particular biological pathways controlling IL-1β synthesis, allowing for fewer side effects while controlling systemic immunity. As a result, upstream targeting can be especially effective if it is carried out at the localised site of inflammation, such as an atherosclerotic plaque. Selective targeting of atherosclerosis by nanoparticle-based agents is therefore being pursued [40].

Despite the fact that most studies only focus on nanocarriers as delivery systems for specific therapeutics to be delivered to atherosclerotic plaques, recent research has shown that nanocarriers with inherent anti-inflammatory and antioxidant activities can be potentially effective next-generation therapies for the treatment of atherosclerosis and other inflammatory conditions [42]. The injured vascular endothelium is known to secrete cell adhesion molecules (e.g., intercellular cell adhesion molecule-1, P-selectin, and E-selectin) in order to attract monocytes throughout the course of atherosclerosis. This leads to the creation of foam cells, which then draw in additional monocytes by secreting interleukin-6 (IL-6), thereby enhancing the synthesis of chemokines (e.g., monocyte chemoattractant protein-1 [MCP-1] and CXC chemokine receptor by the ECs [43,44]. As a result, inflammatory mechanisms are responsible for the pathophysiology of atherosclerosis [41,45].

Atherosclerosis is characterised by the thickening and hardening of arterial walls and narrowing of the lumen. The haemodynamic behaviour is altered by varying degrees due to rupture of an atherosclerotic plaque, which causes thrombosis and can ultimately result in ischaemic strokes and MI [46]. Extensive research is being conducted on targeted drug delivery using microparticles (MPs) and NPs, both of which have significant potential for usage [47]. However, designing suitable carriers that could provide an adequate density of particle-targeted site contact while having high adhesion qualities, and the capacity to migrate towards arterial walls present a significant problem. Furthermore, the distribution of carriers inside the vessel cross-sections and NP characteristics, viz., size, shape, and blood flow factors, such as haematocrit and vessel size, are crucial for the drug carrier design and the successful delivery of micro- and nanocarriers [48]. 

Nanosystems for the treatment of atherosclerosis under investigation have yielded impressive outcomes, mostly based on polymer NPs that were employed as DDS [49,50]. Due to their great biocompatibility, lipid NPs, including liposomes and HDL-like NPs, have also been utilised as DDS over the past ten years [51,52]. These NPs can also be employed for multimodal imaging when combined with dyes or contrast agents made of NPs [53,54]. Nanotechnology allows for the reduction of off-target drug cyto-toxicity, improvement in drug solubility, reduction in dosage requirements, and the capability of nanotheranostic agents that combine diagnostic and therapeutic agents to treat atherosclerosis, as well as the accumulation of agents at specific sites [55]. Active targeting and passive targeting are two different types of target DDS [56]. To treat atherosclerosis, NP innovation has improved active targeting, plasma half-life, and vascular margination. As a result of their form, complex surface interaction, and flexibility, platelets can marginate to the arterial walls and selectively engage with vascular sites of damage [32,57]. The familiar physiological term “margination”, characterises the lateral drift of leukocytes towards endothelial walls [58,59]. Similar to how a marginating particle is one that prefers to migrate near lumen walls, margination dynamics is the movement of the particle laterally from the blood vessel core to the walls [59]. The propensity of red blood cells to orientate themselves centrally within a blood vessel and leukocytes to drift toward vessel walls, are both patterns that are controlled by various active physiochemical processes, although the motion of inorganic deliverable particles can be primarily explained by the shape and morphology of the particles. Additionally, it should be highlighted that while physiological margination has nothing to do with gravitational forces or other similar external fields, synthetic particle margination is greatly influenced by these fields and could be managed in the same way [58].

**Figure 4:**
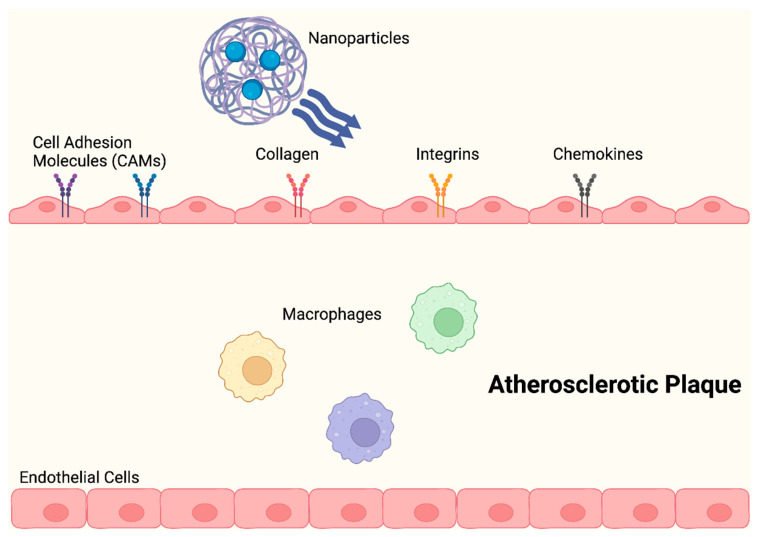
NPs change the shape of atherosclerotic plaques by significantly lowering proinflammatory cytokine levels and preventing macrophage proliferation, during the early stages of atherosclerosis.

Anselmo et al. (2014) described NPs that had platelet-like properties, including signs of damage, with injury-specific aggregation being amplified by injury-specific adhesion and margination. As opposed to those with spherical and stiff discoidal morphology, these platelet-like NPs (PLN) with discoidal morphology and mechanical flexibility, showed improved surface binding. Additionally, under physiological flow conditions ex vivo, site-selective adhesive and platelet-aggregatory characteristics were established. Studies conducted in vivo revealed that PLN accumulated at the injury site and reduced bleeding time by 65%, thereby successfully imitating and enhancing the haemostatic properties of natural platelets [32,57]. In order to aid nanocarriers in evading the local immune system, numerous biomimetic design ideas have arisen. During the early stages of atherosclerosis development, an activated endothelium expresses increased levels of adhesion molecules and chemokines, which encourage the recruitment of leukocytes (Figure 4) [60].

Rapamycin was very recently loaded onto leukosome nanoplatforms. These biomimetic nanocarriers altered the shape of the plaque by drastically reducing proinflammatory cytokine levels and inhibiting macrophage growth [61]. Activated ECs may also accumulate macrophage membrane-coated NPs carrying rapamycin cargoes, which effectively stop the progression of atherosclerosis and macrophage phagocytosis in vivo [62]. Platelets exhibit a significant affinity for atherosclerotic plaques through a variety of mechanisms, including adhesion and aggregation. On the basis of this, Song et al. (2019) created PNPs that integrated rapamycin, which could target and stabilise atherosclerotic plaques [63]. The RBC membrane has also been used to cloak NPs since it has a greater EPR, longer half-life, and higher biocompatibility [64,65]. The progression of atherosclerosis could also be slowed down by using red blood cell membrane-coated PLGA to specifically administer rapamycin to atherosclerotic plaques [66]. Local drug delivery with the help of nanomaterials may exhibit improved clinical outcomes as compared to systemic drug delivery.

Nanocarriers could avoid restenosis following post-balloon angioplasty and reduce the incidence of late adverse events associated with DES, including late stent thrombosis [67,68,69,70]. In order to distribute rapamycin to inflamed regions, a pH-sensitive and ROS-responsive *β*-cyclodextrin (Ox-bCD) nanoplatform was created [71,72]. The release of rapamycin from these nanoplatforms that respond to two stimuli could enable passive targeting of the damaged vasculature and inhibit vascular smooth muscle cell (VSMC) proliferation and migration. The arterial restenosis rat model experiment demonstrated that an intravenous (IV) injection of rapamycin-loaded Ox-bCD NPs could significantly reduce neointimal hyperplasia. Since then, Zhu et al. (2017) have created bilayered NPs with the capacity to successively release inflammatory protein pentraxin 3 (PTX3) from the core and plasmids expressing vascular endothelial growth factor (VEGF) from the outer layer [73]. These bilayered nanomedicines were locally injected with balloon angioplasty to demonstrate quick endothelial regeneration and restenotic inhibition [74].

An intriguing method of actively targeting atherosclerotic plaques was created and described by Kheirolomoom et al. (2015). It relies on cationic lipoparticles (CCL), with anti-miR-712 in the core, and a neutral coating embellished with the peptide (VHPKQHRGGSKGC) to target vascular cell adhesion molecule 1 (VCAM-1). When anti-miR-712, an inhibitor of key pro-atherogenic miRNA, was successfully administered to inflamed mouse aortic ECs in vivo and in vitro, optimal imaging demonstrated that the accumulation was disease-specific. Additionally, atheroma development in a rat model of atherosclerosis was inhibited by a dose of VHPK-CCL-anti-miR-712 that was 80% lower than naked anti-miR-712 [75]. Of late, anti-miR-712 is being employed as a carrier for gold nanospheres (GNS) containing VCAM-1-binding peptide (VHSPNKKGGSKGC) targeted at inflamed endothelia, to prevent the development of atherosclerotic plaques [76].

Recently, the development of anti-atherosclerosis medicines has turned its attention towards inflammation of the endothelium, and various medications have been created to treat hyperlipidaemia and/or inflamed ECs. By blocking 3-hydroxy-3-methylglutaryl (HMG) CoA reductase, statin-related medications, such as atorvastatin, simvastatin, and lovastatin, or curcumin (Cur), a natural polyphenol with anti-oxidation and anti-inflammatory abilities, for instance, have been used to lower plasma lipid levels [77]. Delivery systems that respond to stimuli are also beginning to appear. These systems are based on a variety of triggers, including pH changes, temperature changes, light exposure, reactions to redox potentials, and the application of electric or magnetic fields [36]. At the same time, the localised delivery of NPs via stents can be a successful method for preventing restenosis, by offering consistent drug release in the artery’s target zone [78]. DES can be utilised to localise drug distribution and prevent any possible harm brought about by systemic drug administration [36]. Drug administration by polymer NPs has been utilised to treat restenosis following percutaneous coronary intervention (PCI) [32].

An NP delivery system is ideal for the treatment of restenosis because it may be used for local or targeted distribution, reducing systemic toxicity and reaching specific cell types in sufficient concentrations for the required amount of time. Additionally, inflammatory reactions are not triggered by biocompatible lipids and polymers [35]. NPs that are used to prevent restenosis include liposomes, dendrimers, micelles, polymeric NPs, gel-like NPs, magnetic NPs (MNPs), and quantum dots, amongst many others [79].

#### 2.1.1. Liposomes for Drug Delivery

Liposomal delivery is used in the treatment of CAD [79]. Lipid NPs or liposomes are nano-sized vesicles with spherical shapes, heterogenous sizes, and a lipid-bilayers made of cholesterol, often ranging from a couple hundred to thousands of nm in diameter [27,80]. When delivering hydrophilic drugs in the aqueous core and hydrophobic drugs in the lipid environment, the characteristics of liposomes, such as biocompatibility (due to the use of naturally occurring biologically safe lipids), nm size, and the ability to tailor hydrophilicity and hydrophobicity, could enhance specificity to tissues [81]. Liposomes have a multi-layered structure that enables the use of a single liposomal formulation as DDS. They are effective carriers for delivering genes, stem cells, and anti-inflammatory or antiangiogenic drugs to the site of plaque formation. Liposomes reduce LDL cholesterol levels and have also been utilised in the development of vaccines targeting atherosclerotic mediators [82].

As a result of conjugation with polyethene glycol (PEG)ylation, which prolongs drug circulation time, liposome encapsulation has the advantage of well-established in vivo behaviour. The capacity of liposomes to encapsulate numerous MNP cores and deliver them all at once to a target spot without dilution is another advantage. The ability of these DDS to perform many tasks is further increased by including a medicinal drug in the payload. Similar to this, MNPs have also been entrapped using multifunctional micelles produced by using amphiphilic block copolymers for these applications [83,84]. The ability of peptide-modified liposomes to deliver therapeutic and diagnostic substances to specific blood vessels has been investigated. In order to show the critical function of activated platelets in atherogenesis, the evolution of atherosclerotic lesions, and thrombosis in vascular disorders, Srinivasan et al. (2010) added ligands that bind to surface receptors on activated platelets (e.g., P-selectin and integrin GP IIb/IIIa) and liposomes [85]. Moreover, according to the Phase 1 results of a study conducted by Gutman and Golomb (2012), liposomal alendronate can lower in-stent restenosis (ISR) to 40.1%, compared to 73.5% in empty liposomal alendronate, after 28 days of follow-up in a rabbit model carotid artery [86].

Currently, pharmacological therapies for atherosclerosis frequently focus on either controlling cholesterol levels, or managing inflammation to slow down disease progression. However, they are unable to directly dissolve atherosclerotic plaques and reverse atherosclerosis, in part because the drug does not accumulate well in the atherosclerotic plaques. Gao et al. (2022) created a macrophage–liposome conjugate delivery system, through a host–guest interaction to enhance macrophage recruitment for the targeted treatment of anti-atherosclerosis, using synergistic plaque lysis and enhanced anti-inflammatory effects. Endogenous macrophages are used as drug-transporting cells, after having their membranes modified with a β-cyclodextrin (β-CD) derivative to create macrophages with β-CD decorations (CD-MP). Through host–guest interactions between β-CD and ADA, adamantane (ADA) modified quercetin (QT)-loaded liposome (QT-NP) can be attached to CD-MP, to create a macrophage–liposome conjugate (MP-QT-NP). As a reactionary action to inflammation of the plaque, macrophage transports liposome conjointly to significantly enhance the build-up of anchored QT-NP in the aortic plaque. On top of the anti-inflammatory properties of QT, MP-QT-NP effectively regresses atherosclerotic plaques from both rodent and human carotid arteries via CD-MP-driven cholesterol efflux, since excess membrane β-CD is known to bind to cholesterol. Transcriptome analyses of atherosclerotic rodent aorta and human carotid artery tissues, have shown that MP-QT-NP may upregulate the liver X receptor to promote cholesterol efflux, while simultaneously activating the NRF2 pathway to minimise plaque inflammation [87].

In a study by Li et al. [88], arsenic trioxide (Ato) and Cur were co-delivered by liposomes modified with a targeting ligand (E-selectin-binding peptide), to dysfunctional ECs that overexpressed E-selectin. Using apolipoprotein E (apoE) knockout (ApoE−/−) mice, the anti-atherosclerosis properties of liposomes co-loaded with Ato and Cur were examined in vivo. Ato and Cur were administered to defective ECs using targeted liposomes, which together suppressed adhesion molecules and plasma lipid levels. Moreover, by preventing monocyte migration into the intima; this therapy decreased foam cell production and the release of inflammatory factors. Cur also effectively decreased Ato-inducible cytotoxicity [88], reflecting the effectiveness of liposomes as a delivery system for atherosclerosis. Chono et al. (2005) examined the drug delivery and anti-atherosclerotic impact of dexamethasone-liposomes (DXM-liposomes) to atherosclerotic lesions in atherogenic mice, so as to validate the effectiveness of liposomes incorporated with dexamethasone (DXM) as treatment for atherosclerosis [89]. To further understand how particle size affects the delivery of drugs to atherosclerotic lesions, and how that affects atherosclerosis, the same investigators used three distinct particle sizes—68.6 nm (L70), 202 nm (L200), and 519 nm (L500). Mice were then fed an atherogenic diet for 14 weeks before being utilised as an animal model to develop atherosclerotic plaques in their aortas. The IV injection of DXM-liposomes to atherogenic mice, allowed researchers to study their aortic pharmacokinetics. When compared to animals treated with L500, L70, or free DXM (f-DXM), atherogenic mice treated with L200 had an aortic uptake clearance of DXM that was 2.6- to 3.2-folds higher. A measurement of the aortic cholesterol ester levels was used to assess the anti-atherosclerotic effects of DXM-liposomes. In comparison to animals treated with phosphate-buffered saline (PBS), atherogenic mice administered L200 (55 μg DXM/kg) had considerably decreased aortic cholesterol ester levels. As L200 had a substantially stronger anti-atherosclerotic effect than f-DXM, these results imply that L200 could effectively transport DXM to atherosclerotic lesions, resulting in a potent anti-atherosclerotic response at a lower dose. L200 may therefore be helpful in the creation of DDS for the treatment of atherosclerotic disease. Haematoxylin and eosin stained of transverse sections of the aortic root of animals in the control group showed foam cell infiltration and extensive cholesterol clefts, which are the typical signs of atherosclerotic lesions. On the other hand, animals treated with liposomes showed few foam cells and intimal structures that appeared generally normal. These findings revealed that the combination of Ato and Cur administered by EC-targeted liposomes culminated in increased anti-atherosclerotic effects [89]. The findings of a study by Danenberg et al. (2002) demonstrated that systemic administration of the liposomes as opposed to local administration, resulted in the greatest reduction in neointimal growth, which was probably related to the drugs’ mode of action [90]. These medications affect macrophages, which are crucial to the inflammatory process leading to restenosis [91]. The hydrophilic bisphosphonates are being introduced into macrophages via liposomes, which is a unique drug delivery strategy for this class of compounds [35].

Lobatto et al. (2010) investigated the anti-inflammatory properties of steroid-loaded liposomes in an atherosclerosis-causing rabbit model. T_1_-weighted magnetic resonance imaging (MRI) was used to track the transport of drug-loaded liposomes, and dynamic contrast-enhanced MRI (ceMRI), positron emission tomography (PET) and histological evaluation, were used to assess the effectiveness of the therapy [92]. In this same study, long-circulating liposomes were demonstrated to be a highly effective drug delivery vehicle to atherosclerotic plaques, using both in vivo and in vitro imaging. Liposomal proteolipid protein (PLP) injection has a significantly higher therapeutic efficacy than free-circulating PLP, for which no appreciable alterations in the inflammatory state of the atherosclerotic lesions were found. There was a measurable number of liposomes in the atheroma. Angiogenesis and increased capillary permeability are features of atherosclerotic lesions [92]. The EPR effect is responsible for the technique of focused distribution and accumulation of liposomes in the atheroma. A PEGylated liposomes is an example of a long-circulating macromolecular substance that extravasates from the bloodstream and builds up at sites of inflammation, where it is maintained and act locally [93]. Due to the targeting effect, the drug is delivered to the target site more effectively, allowing for the lowering of drug dosages while maintaining the same level of efficacy. A higher percentage of the drug dose administered intravenously in the liposome-encapsulated form, in comparison with the non-liposomal form, will end up in the plaques because of the long-circulating nature of liposomes. Targeting ligands can be conjugated to the liposomal surface to deliver drugs to locations with increased capillary permeability, such as angiogenic ECs, in addition to passively targeting liposome-encapsulated glucocorticoids. A number of investigations have demonstrated that liposomes can be employed as drug-targeting molecules on the surfaces of entities of interest [80].

#### 2.1.2. Dendrimers for Drug Delivery

The shear stress that atherosclerosis exerts is more than 100 times greater than that found in healthy blood arteries. Vascular inflammation encourages the creation of excessive reactive oxygen species (ROS) [94]. The combination of high shear stress and an elevated amount of ROS at the plaque creates a unique microenvironment, that can be leveraged to create a delivery system that is both ROS- and shear stress-responsive [95]. Shen et al. (2021) reported the first shear stress-responsive NP for the in vivo delivery of SV, using dendrimers in the treatment of atherosclerosis [96]. Dendrimers are made up of a single molecule that has an inner core that was created initially and a succession of macromolecular branches that were added in stages using discrete units. They have a special structure for drug delivery applications since they can exhibit functional groups in multiple copies on their surface [97]. SA PAM exhibited the ability to detach from the RBC surface when exposed to shear stress. The efficacy of SA PAM@RBCs was evaluated using both the FeCl_3_ and ApoE^−/−^ models, with results showing superior therapeutic effects compared to free SA. In vivo studies demonstrated the excellent safety of SA PAM@RBCs [96]. In recent years, there has been an increasing preference for cationic polyamidoamine (PAMAM) dendrimers in CAD treatments [98]. PAMAM dendrimers, which were the first commercial dendrimers with structures resembling those of proteins, had distinct dendritic structures and three-dimensional spherical shapes. PAMAM dendrimers have been employed extensively in the field of biomedical nanotechnology because of their excellent water solubility, monodispersity, tuneable molecular size, non-immunogenicity, capacity to traverse biological barriers, and ease of functionalisation [99,100,101]. PAMAM zero generation (G0) dendrimers were investigated as nanocarriers for drug administration, and conjugated G0 PAMAM dendrimers incorporated with a zinc phthalocyanine (ZnPc) photosensitiser were investigated for their effects on the sick and healthy tissues removed from human carotid arteries. The nanocarriers had different levels of affinity for atheromatous tissue and healthy tissue, as revealed by atomic force microscope (AFM) images using fractal analytical techniques and characterisation with Minkowski functionals (a mathematical method for functional analysis). There were noticeable differences in the aggregation behaviours of G0 and G0/ZnPc nanomaterials. There were reports of larger G0/ZnPc aggregation on the atheromatous plaque [98]. The abovementioned findings suggest that a biomimetic DDS, with dual responsiveness to ROS and shear stress, holds great promise as a potential strategy for treating atherosclerosis.

#### 2.1.3. Micelles for Drug Delivery

Micelles are created when amphiphilic molecules go through self-assembly, because the hydrophobic sections of the molecules, group together to form the interior which minimises energy. Long circulation times are made possible by the hydrophilic shell, which also accounts for the greater stability in vivo. It is possible to encapsulate and transport either bioactive medicinal compounds, diagnostic agents, or both using the hydrophobic core of micelles. Attaching moieties, such as targeting ligands, to the outer layer of the micelle can lead to a higher level of active binding to cells and tissues relevant to the disease [81]. According to some findings, phospholipid-based micelles have stronger anti-restenotic effects than PEGylated liposomes, which is likely because phospholipid-based micelles are noticeably smaller than the PEGylated liposomes [102,103]. Based upon the block copolymer of poly(ethylene glycol) and poly(propylene sulphide) (PEG-PPS), Wu et al. (2018) constructed a smart DDS using andrographolide-loaded micelles that react to the oxidative microenvironment of atherosclerotic plaques, with the goal of simultaneou-sly reducing inflammatory responses and ROS levels in treating atherosclerosis [104]. As PEG-PPS is ROS-responsive, the micelle not only functions as a stimuli-responsive drug carrier to rapidly release the drug andrographolide from its capsule but it also consumes ROS on its own at the pathological sites, effectively suppressing the expression of pro-inflammatory cytokines and reducing oxidative stress. As a result, the andrographolide-loaded micelle showed extraordinary therapeutic effects both in vivo and ex vivo. In sum, andrographolide-loaded PEG-PPS micelle offers a promising and cutting-edge approach against atherosclerosis by synchronically reducing oxidative stress and inflammation [104].

Shen et al. (2021) developed a DDS using simvastatin-loaded micelles (SV MC) @RBCs, with a dual responsiveness to ROS and shear stress [105]. This DDS effectively releases the drug SV in the presence of ROS, offering targeted therapy while minimising the risk of bleeding associated with SV administration. SV MC@RBCs effectively inhibit macrophage uptake and prevent systemic clearance, leading to enhanced drug retention. The controlled release of SV at specific sites is achieved through the stimuli-responsive nature of the system, triggered by ROS and high shear stress. SV MC contributes to the reduction of cellular oxidative stress, resulting in a synergistic therapeutic effect. The SV MC@RBCs DDS hence demonstrates remarkable therapeutic efficacy in the treatment of atherosclerosis, while maintaining excellent safety within the effective dosage range [105].

#### 2.1.4. Polymeric Nanoparticles for Drug Delivery

Polymeric NPs can be constructed in various forms, including solid, hollow, and dense porous structures, Examples of porous structures are nanospheres and nanorods, while nanoshells and nanocapsules are examples of hollow structures [35,106]. DES used in PCIs are attributed to a considerable decrease in artery restenosis, because they effectively combine a mechanical scaffold with a DDS [107]. Even though DESs are frequently used in clinical settings to maintain vascular patency, not all coronary stenosis can be treated with DES insertion [108]. DESs also need prolonged antiplatelet therapy, since they are linked to delayed endothelialisation and increased thrombogenicity [69,109,110,111]. In a study conducted by Uwatoku et al. (2003), doxorubicin, an antiproliferative medication, was included in polymer nanosystems made of the core-shell NPs of polyethyleneglycol-based block copolymers, which were then delivered to the balloon-damaged artery [50]. In another study by Nakano et al. (2009), DDS using polymer NPs were also applied to the stent surfaces using biodegradable PLGA NPs that included the fluorescent market fluorescein isothiocyanate (FITC). The stainless steel balloon-expandable stents were coated with this drug delivery nanosystem. This NP-eluting stenting system demonstrated distinct qualities of innovative vascular compatibility, electrodeposition coating technology, and sustained transport of the FITC marker into the stented swine coronary artery, in comparison with the dip-coated polymer-eluting stent [49].

Leal et al. (2022) developed a novel DDS with polymeric NPs that were modified with an antibody capable of binding to VCAM-1, which is overexpressed in inflamed arterial endothelia, resulting in the sustained release of the cargo within the cells. NPs loaded with Ato and miRNA exhibited non-toxicity to cells across a wide range of concentrations, allowing for a significant reduction in the levels of proinflammatory cytokines, IL-6 and TNF-α, as well as ROS in both LPS-activated macrophages and vascular ECs. The dual-loaded NPs demonstrated superior prevention of LDL accumulation within macrophages, and greater preservation of cellular morphology compared to the single-loaded NPs [112].

Chan et al. (2011) demonstrated the localisation of a focused NP DDS, known as nanoburrs, to damaged vasculature, reflecting the effectiveness of using paclitaxel-encapsulated nanoburrs as a DDS to suppress stenosis in injured vessels [113]. Heptameric peptides were identified and isolated from an M13 bacteriophage screen against collagen IV and functionalised using PLGA and PEG polymer core-shell interfaces to create the nanoburrs [114,115,116]. In a rat carotid injury model, the systemic treatment of fluorescently labelled nanoburrs increased nanoburr localisation to angioplastied arteries by 50% compared to nontargeted NPs and by threefold compared to healthy arteries [114]. Taking into account the recent advancements in DES design and long-term clinical safety studies, nanoburrs could be helpful for a portion of individuals who are unable to obtain DESs [117,118,119]. This group of individuals may have comorbidities that have already developed, scheduled procedures, or lesions that cannot be stented (such as diffusely diseased and smaller arteries, longer lesions, and branch points) [108]. The nanoburrs may eventually be employed in conjunction with DESs or BMSs in patients who have already received them to retain long-term patency. Hence, they may have clinical utility in procedures such as plain old balloon angioplasty (POBA). Prestenotic lesions may also be treated with anti-inflammatory drugs using nanoburrs in early-stage CAD [120]. Additionally, the extended circulatory half-life of polymeric NPs may improve retention at areas with increased permeability [119]. In addition, the ability to surface functionalise NPs allows for the addition of targeting ligands other than peptides, such as small molecules and antibodies, to enhance drug delivery to regions of vascular damage [121]. The results suggest that systemically delivering nanoburrs as a therapy for damaged vasculature has clinical advantages. Independent of artery anatomy or stent installation, this NP-based therapy achieves its efficacy through effective vascular targeting and controlled drug release [113].

#### 2.1.5. Gel-like Nanoparticles for Drug Delivery

It was previously shown that ECs and VSMC ingested hydrogel nanospheres (100 nm) constructed of poly (N-isopropylacrylamide) more thoroughly than microspheres, albeit intracellular uptake was reliant on nanosphere concentration, incubation duration, and applied shear stress levels in the medium [122]. On the other hand, microspheres were quickly ingested by phagocytes, particularly in high quantities. These results imply that in terms of vascular absorption and biocompatibility, hydrogel nanospheres were a more efficient intravascular DDS in the treatment of atherosclerosis [122]. Given the fact that a major portion of VSMC quickly undergoes apoptosis after balloon angioplasty, Reddy et al. (2008) hypothesised and explored the idea of preventing VSMC from going through apoptosis to stop intimal hyperplasia. Rapamycin, which had anti-proliferative and anti-apoptotic properties, was loaded into gel-like NPs. There was a considerable reduction of hyperplasia and better re-endothelialisation of the wounded artery after infusion in the rat carotid artery injury model. It was also reported that the caspase 3/7 enzyme systems were inhibited in the treated artery, which prevented the apoptosis of VSMCs [123].

#### 2.1.6. Magnetic Nanoparticles for Drug Delivery

Researchers have been interested in the active drug delivery approach of magnetic drug targeting (MDT) for use in regenerative medicine, tissue engineering, gene therapy, directed cellular activity, and magnetic imaging. Different techniques have been used to produce drugs coated on magnetisable particles, such as iron oxide, in a variety of sizes [124]. NP DDS that are mechanically responsive can be propelled by magnetic fields, ultrasound, or shear forces [125]. Only magnetically responsive nanoplatforms, for example, superparamagnetic iron oxide NPs (SPIONs) and contrast agents based on gadolinium (Gd) are capable of receiving force from the magnetic field when it is applied remotely. To aid in drug release, these magnetic NPs are typically coupled with other responsive and flexible materials when an alternating current magnetic field is present [126]. By utilising magnetic field-directed magnetic vehicles for specific transportation within blood vessels, anti-ISR therapies, cell, gene, therapeutic protein delivery, and the stented artery delivery of paclitaxel have all been suggested as potential benefits of MDT. MDT shows great potential in accurately directing therapeutic agents to areas affected by CAD, while reducing the risk of unintended distribution (thereby addressing safety concerns), and enabling the precise and long-lasting targeting of deep tissues [127].

Primary ECs that were loaded with functional MNP were used as vectors to target vascular stents, increased EC growth and survival-related gene expression, inhibited EC gene-related coagulation, suggested re-endothelialisation by the implant, and decreased neointimal hyperplasia [128]. Iron-, cobalt-, or nickel-based metallic MNPs are frequently created using a core-shell structure, with gold or silica used as the coating material. A tightly packed cubic lattice is formed by iron MNP made of nanocrystalline magnetite (Fe_3_O_4_) or maghemite (γFe_2_O_3_). Additionally, gold shell NPs (~120 nm) have been utilised in imaging and therapeutic applications [129]. To take advantage of nanoscale magnetic phenomena such as enhanced magnetic moments and superparamagnetism, a variety of MNPs with distinct molecular compositions have been developed, and studied for biomedical applications. Advances in nanotechnology now make it possible to precisely engineer the essential properties of these tiny particles, just like other nanomaterial-based systems. It is possible to modify the composition, size, shape, and surface chemistry of NPs to influence their in vivo behaviour, as well as their magnetic properties [130,131]. A biomedical MNP platform in its most basic form has a code made up of inorganic NPs, and a surface layer that is biocompatible, providing stability in physiological conditions. The incorporation of useful ligands is additionally made possible using appropriate surface chemistry [132]. Due to its modular construction, MNPs may carry out several tasks at once, including drug delivery, multimodal imaging, continuous monitoring, and combined therapeutic techniques [133].

The behaviour of these MNP DDS in vivo poses a considerable obstacle to their applications. A major limitation to the efficacy of several of these systems is their inability to cross the blood–brain barrier (BBB) or vascular endothelium, and their identification and removal by the reticuloendothelial system (RES), before they can reach the intended tissue. Upon IV administration, the fate of the MNPs is greatly influenced by their morphology (size and shape) and surface chemistry (charge and hydrophobicity). These physicochemical characteristics of NPs have a direct impact on their pharmacokinetics and biodistribution [134]. Several strategies have been used to promote the effectiveness of MNPs. For instance, reducing their size and attaching non-fouling polymers to enhance their stealth properties, and prolonging their circulation in the bloodstream, to increase the likelihood of reaching the intended areas [135,136]. New types of nanocrystalline cores, functional ligands, and coating materials are being incorporated into the latest generation of MNP-based contrast agents for MR imaging and carriers for DDS. These enhancements aim to improve the detection and targeted distribution of these NPs. High magnetic moments are provided by new MNP core formulations, such as metallic and alloy NPs, doped iron oxide nanocrystals, and nanocomposites, which improve the signal-to-background ratios during MRI. Novel surface coatings, such as durable gold or silica shell structures, allow for the application of hazardous core materials, as well as a more complete coating, by facilitating the formation of self-assembled monolayers (SAMS) on the NP surface [137,138].

The asymmetric bifurcation-driven steering of magnetic NPs was the subject of a publication by Cherry and Eaton (2014), who developed a model to illustrate how the three variables, viz., haematocrit, shear rate, and applied magnetic force impacted the three-dimensional movement of ION clusters [139]. They concluded that the particles must be clustered together to achieve adequate magnetic displacement, and during bifurcation the best approach is to apply equal magnetic gradients (up to 10 T/m) towards the branch, and opposite to the streamwise direction [139]. In a study conducted by Haverkort et al. (2009), a comparable approach was utilised to simulate MDT in a configuration that resembled the left coronary artery (LCA) based on angiographies from healthy individuals [140]. They integrated a superconducting cylindrical magnet that was placed in different orientations and was located at a distance from the patient’s chest that closely resembled the distance between LCA and the chest, which was approximately 5 cm. Their model also used very large iron–carbon particles of 0.25–4 μm. Depending on where the magnet is placed, the LCA model’s capture efficacy was observed to rise linearly with the particle diameter. The calculations and outcomes presented provided convincing grounds for additional MDT investigations, including the integration of physiological or clinically relevant parameters, such as the magnet’s orientation or position in relation to the target tissue. This is despite the fact that their models contained several simplifications, such as limited fluid dynamics, that may underestimate the capture efficacy of smaller particles [140].

There were a few interesting results from a study conducted by Badfar et al. (2020), which explored MDT employing drug-coated Fe_3_O_4_ NPs to the stenosed region of the conduit, with the use of a magnetic wire source. The drug follows the base fluid flow in the absence of a magnetic field, and a minute amount of the drug is present on the bottom wall of the stenosed region as the target tissue. The progressive development of drug droplets on the position of the wire is the most significant occurrence in low magnetic numbers MnF = 16.4. The drug droplet plays a beneficial role in the MDT because it keeps the drug close to the target tissue for an extended period. Due to the increased kelvin force in moderate magnetic numbers MnF = 32.8 and 65.6, a significant amount of the drug builds up on the wire location and generates a larger drop. The amount of drug in the target tissue area rises swiftly to its maximum at the instant the droplet forms in the magnetic number MnF = 164. Another feature seen in MnF = 164 is the creation of vortices in the tissue area. Vortices disrupt the MDT and cause the medication to diffuse away from the intended target tissue. The amount of drugs delivered to the tissue at the time the droplet forms are also high for high magnetic numbers of MnF = 328 and 656. However, when more vortices are created in the flow, the drug is forced further away from the tissue. Thus, it can be concluded that the magnetic number MnF = 164 is associated with the ideal performance of MDT. The positive impact of magnetophoresis is strong in this magnetic number, while the negative impact of vortex formation is weak. The performance of MDT is not considerably impacted by the wire’s position. The outcomes of this investigation demonstrated that the MDT approach may effectively transport drugs to vessels with stenosis in atherosclerotic patients [141].

Kashan and Haik (2006) investigated MNP carriers with polyvinyl alcohol (PVA) coatings in a tube with a magnetic field [142]. On MDT, they observed into MNP size, shape, and distance from the tube wall. NPs have a tendency to creep down vessel walls close to magnets, and before target areas when subjected to the magnetic force of a permanent magnet field [142]. Akbar (2016), examined the MNPs to determine how blood flowed through the stenosed, tapered channel [143]. Nadeem and Ijaz (2016) also analysed the impact of a magnetic field on stenosed non-laminar vessel flow [144]. Providing adequate magnetic gradients to steer a considerable quantity of nanocomplexes, even against the blood flow, is the key unresolved hurdle for clinical MDT methods, aside from drug release problems. When the cardiovascular system and its associated high arterial flow rates are involved, the problem becomes considerably more challenging. For comparison, magnetic attraction forces are often outnumbered by hydrodynamic drag forces in big vessels. Simple handheld magnets will therefore be ineffective beyond animal models. However, for future MDT applications, approaches combining ION-loaded cells/vehicles with optimised Halbach arrays, configurations of electromagnets, or clinical MRI scanners offer interesting options going forward [145].

#### 2.1.7. Quantum Dots for Drug Delivery

Monocytes implicated in inflammatory activation have a pathogenic cause for the rupture of atherosclerotic plaque, according to an increasing body of experimental and clinical evidence [43]. Continuous mononuclear cell infiltration is typically regarded as the first stage in the formation of atherosclerotic plaque. MCP-1 mediates the ongoing recruitment of monocytes to the atherosclerotic plaque during this phase, which is triggered by endothelial shear stress (ESS) [146,147]. Since monocytes can access the interior of the plaque, they are obviously the perfect gene delivery vector. However, it is difficult to directly load miRNAs onto the surface of monocytes. An intermediary gene delivery vector is required to overcome the constraints of monocytes. Although successful in mammalian cells, viral gene delivery vectors are constrained by biosafety issues [148]. Graphene-based nanomaterials used for gene plasmid delivery represent an area of interest in this regard. Excellent gene loading properties and stable interactions with diverse molecules are provided by π–π-stacked nanoscale graphene. The minute size of graphene quantum dots (GQDs) facilitates easy cellular uptake and reduces cytotoxicity. Liu et al. (2019) created a new gene delivery system utilising GQDs-miRNA through the surface engineering of monocytes. Treating macrophages with gene regulators to inhibit plaque formation proves to be an effective approach in reducing the risk of plaque rupture. In this same study, the injection of engineered monocytes with modified cell function in vivo, revealed an effective reduction of plaque inflammatory reactions and plaque formation. Given these outstanding qualities, GQDs are anticipated to be suitable intermediate gene delivery vectors in the nanotheranostics of atherosclerosis. However, the measurement of miR223 concentration or retention in atherosclerotic plaques was not possible in this study. Additional investigations are necessary to determine the appropriate time interval for IV administration to achieve sustained regulation [149]. Quantum dots have been employed as fluorescent labels to create traceable NP because of its tuneable physicochemical properties, strong photostability, broad absorption spectrum, and relatively narrow emission bands. For example, as the processes of angiogenesis and vascular remodelling, as well as the infiltration of macrophage cells into artery tissues, have all been linked to disease, quantum dots have been proposed to monitor these phenomena [150].

#### 2.1.8. Nanorobots for Drug Delivery

The knowledge gathered from convergent fields such as molecular biology, mesoscopic/supramolecular chemistry, and mesoscopic physics at the nanoscale scale, provides significant advantages for diagnosis and treatment when compared to traditional methods through nanorobotics. Nanorobots can be used to locate atherosclerotic lesions in stenotic vessels, meaning that nanomachines could play a direct mechanical or chemical role in the treatment. To locate the target area, avoid infection, and aid in the healing of inflamed tissues, the robots can also be pre-loaded with a contrast or therapeutic agent. Anti-inflammatory medications have been pay-loaded in biomimetic peptide by polymeric nanoengineered collagen type IV particles with an amino-terminal peptide group created by encircling amino acids into the polymeric structure [151]. Mice used in in vivo experiments show that the plaque was stabilised and the injured arteries were significantly repaired [152]. In addition to collagen particles, a broad variety of polymers can be used to create polymeric nanoproducts for the treatment of atherosclerosis. These include amphiphilic molecules based on poly(ethylene glycol) or poly(ethylene oxide); poly[N-(2-hydroxypropyl)methacrylamide] molecular assemblies or NPs, which are typically based on metal, polymer, or lipid; and block copolymers based on poly(styrene), poly(meth)acrylates, poly(lactic acid), poly(butadiene), poly(propylene oxide), and poly(caprolactone) [55,151]. Table 1 presents a comprehensive overview of several key studies that highlight the recent progress in utilising nanotheranostics in the treatment of atherosclerosis. These studies outlined the various types of nanocarriers employed and the therapeutic agents utilised and provide insights into their key characteristics, advantages, and disadvantages.

## 3. Limitations of Current Nanotheranostic Platforms in Atherosclerosis and Future Work

More research is needed to understand the biological behaviour of NPs, including pharmacokinetics, targeting efficiency, and biocompatibility [153]. Additionally, exploring ultra-shear-responsive nanoplatforms that can quickly react to even a tiny change in shear force is still a very difficult task in the case of atherosclerosis, which benefits from enhanced shear stress in the plaque area. To create a new theranostic system, it is also critically important to consider the combination of shear-responsive nanoplatforms with imaging modalities. When designing MNPs, one should take into consideration their control release potential, in addition to their multiple functions as molecular imaging agents and specific targeted drug delivery vehicles. Moreover, microbubbles and nanobubbles have demonstrated excellent theranostic potential in designs that respond to ultrasound. Multifunctionality for microbubble design can be attained by sheathing them in NPs [154]. All of the established mechanoresponsive nanoplatforms require additional functionalisation, primarily to extend the duration of their bloodstream circulation and prevent immune system elimination. Furthermore, polymers are a common supply of material for nanoplatforms. The performance of polymer-based nanoplatforms under shear stress at various stages of atherosclerosis can be predicted using the mechanical properties of polymers. Establishing a link between the quantification of dynamic shear fluctuations and the development of atherosclerotic plaque may have clinical utility [126].

It is anticipated that in the coming years, researchers will be able to move beyond the drawbacks of current NP designs and implement them widely in both clinical practice and research for the detection and treatment of high-risk atherosclerosis patients.

## 4. Conclusions

Although lipid-lowering medications have been shown to be successful in treating atherosclerosis, CADs continue to be the world’s leading cause of death. The development of novel nanotheranostic platforms for the diagnosis and treatment of atherosclerosis has been aided by advances in both innovative nanotechnology and understanding of the pathobiology of the disease. NPs have become one of the most significant developments in DDS based on nanomedicine for the management of atherosclerosis. NPs provide a secure and efficient delivery mechanism for a range of medications, in which their therapeutic utility is typically limited by systemic toxicity or undesired pharmacokinetic characteristics.

## Figures and Tables

**Table 1:** Summary of key studies detailing different therapeutic nanocarriers used in atherosclerosis therapy.

Nanocarrier	Therapeutic Agent	Characteristics	Advantages	Disadvantages	Reference No.
Liposome	PEG-coated	Liposomes have a multi-layered structure that enable the use of a single liposomal formulation as DDS for drugs and contrast agents.	Liposomes are effective carriers for delivering genes, stem cells, as well as anti-inflammatory or antiangiogenic drugs, to the site of plaque formation. Liposomes reduce LDL cholesterol levels and have also been utilised in the development of vaccines targeting atherosclerotic mediators.	The flow of blood in the vessels exerts shear stress on the endothelial wall, which can lead to NPS being washed away from the targeted site and reduce the duration of interaction between NPs and their target within the plaque. There is an urgent need to address the large-scale production of targeted liposomes with various ligands attached to their surface.	[82]
Cross-linked dendrimer NPs	Simvastatin acid (SA)	A biomimetic DDS with dual responsiveness to ROS and shear stress for atherosclerotic treatment was developed, which involved loading SA into cross-linked dendrimer NPs (SA PAM). These NPs were then adsorbed onto the surface of RBCs to create SA PAM@RBCs. This novel DDS was designed to respond to both ROS and shear stress, providing a targeted and controlled drug delivery.	SA PAM exhibited the ability to detach from RBC surface when exposed to shear stress. The efficacy of SA PAM@RBCs was evaluated using both the FeCl_3_ and ApoE^−/−^ models, with results showing superior therapeutic effects compared to free SA. In vivo studies demonstrated excellent safety of SA PAM@RBCs.	-	[96]
Micelles	Simvastatin (SV)	A DDS using SV-loaded micelles (SV MC)@RBCs, was developed with a dual responsiveness to ROS and shear stress. This system effectively releases the drug SV in the presence of ROS, offering targeted therapy while minimising the risk of bleeding associated with SV administration. The SV MC@RBCs DDS demonstrates remarkable therapeutic efficacy in the treatment of atherosclerosis, while maintaining excellent safety within the effective dosage range.	SV MC@RBCs effectively inhibit macrophage uptake and prevent systemic clearance, leading to enhanced drug retention. Controlled release of SV at specific sites is achieved through the stimuli-responsive nature of the system, triggered by ROS and high shear stress. SV MC contributes to the reduction of cellular oxidative stress, resulting in a synergistic therapeutic effect.	-	[105]
Polymeric PLGA NPs	miRNA-124a and statin atorvastatin (Ato)	Polymeric NPs were modified with an antibody capable of binding to vascular adhesion molecule-1 (VCAM1), which is overexpressed in an inflamed arterial endothelium, resulting in sustained release of the cargoes within the cells. Dual-loaded NPs demonstrated the superior prevention of LDL accumulation within macrophages and greater preservation of cellular morphology compared to the single-loaded NPs.	NPs loaded with Ato and miRNA exhibited non-toxicity to cells across a wide range of concentrations, allowing for a significant reduction in the levels of proinflammatory cytokines IL-6 and TNF-α, as well as ROS, in both LPS-activated macrophages and vessel endothelial cells.	-	[112]
Magnetic Fe_3_O_4_ NPs	Unspecified drug (numerical simulation)	A numerical simulation was conducted to study the MDT of Fe_3_O_4_ NPs coated with drugs to the stenosis region using a magnetic field generated by a wire.	Optimal MDT performance is achieved when the magnetic number is around 164, at which the positive effect of magnetophoresis is high, and the negative effect of vortex formation is low.	Vortices negatively impact the MDT process by causing the drug to diffuse outside the intended target tissue.	[141]
Graphene quantum dots (GQDs)	miRNA223	A new gene delivery system utilising GQDs-miRNA is created through the surface engineering of monocytes. Treating macrophages with gene regulators to inhibit plaque formation proves to be an effective approach in reducing the risk of plaque rupture.	In vivo, the injection of engineered monocytes with modified cell function demonstrates the effective reduction of plaque inflammatory reactions and plaque formation.	The measurement of miR223 concentration or retention in atherosclerotic plaques was not performed in this study. Additional investigations are necessary to determine the appropriate time interval for intravenous administration to achieve sustained regulation.	[149]
Nanorobots	Collagen type IV particles	Nanomachines may be directly involved in the treatment process mechanically or chemically, since nanorobots can be used to locate atherosclerotic lesions in stenotic vessels.	Nanorobots can also come pre-loaded with a contrast or therapeutic agent to help them find the target area, prevent infection, and speed up the healing process of inflamed tissues.	The space available for the built-in energy source for efficient controllable propulsion and steering is extremely limited because of the small size of nanorobots. Due to the absence of a proven technology for producing nanorobotic systems, especially for biomedical applications, this domain is still only a dream.	[151]

## Abbreviations

ADA	adamantane
AFM	atomic force microscope
apoB	apolipoprotein B
apoE	apolipoprotein E
ATO	arsenic trioxide
BBB	blood–brain barrier
β-CD	β-cyclodextrin
CAD	coronary artery disease
CCL	cationic lipoparticles
CD-MP	macrophages with β-CD decorations
ceMRI	contrast-enhanced MRI
Cur	curcumin
DDS	drug delivery systems
DES	drug-eluting stents
DXM	dexamethasone
DXM-liposomes	dexamethasone-liposomes
ECs	endothelial cells
ESS	endothelial shear stress
f-DXM	free DXM
Fe_3_O_4_	iron MNP made of nanocrystalline magnetite
FITC	fluorescent marker fluorescein isothiocyanate
G0	zero generation
Gd	gadolinium adamantane
GNS	gold nanospheres
GQDs	graphene quantum dots
HDL	high-density lipoproteins
HLA	hyaluronic acid
HMG	3-hydroxy-3-methylglutaryl
IL-6	interleukin-6
IL-1β	interleukin-1β
ISR	in-stent restenosis
IV	intravenous
LCA	left coronary artery
MCP-1	monocyte chemoattractant protein-1
MDT	magnetic drug targeting
MI	myocardial infarction
miRNAs	microRNAs
MNPs	magnetic nanoparticles
MP-QT-NP	macrophage-liposome conjugate
MPs	microparticles
MRI	magnetic resonance imaging
NPs	nanoparticles
Ox-bCD	ROS-responsive β-cyclodextrin
PAMAM	polyamidoamine
PBS	phosphate-buffered saline
PCI	percutaneous coronary intervention
PDT	photodynamic therapy
PEG	polyethene glycol
PEG-PPS	PEG and poly(propylene sulphide)
PET	positron-emission tomography
PLGA	poly lactic-co-glycolic acid
PLN	platelet-like NPs
PLP	proteolipid protein
POBA	plain old balloon angioplasty procedures
PTX3	pentraxin 3
PVA	polyvinyl alcohol
QT	quercetin
QT-NP	quercetin-loaded liposome
RES	reticuloendothelial system
ROS	reactive oxygen species
SAMS	self-assembled monolayers
SPIOs	superparamagnetic iron oxides
SPIONs	superparamagnetic iron oxide NPs
SPR	surface plasmon resonance
SV MC	simvastatin-loaded micelles
USPIO	ultrasuperparamagnetic iron oxides
VCAM-1	vascular cell adhesion molecule 1
VEGF	vascular endothelial growth factor
VSMC	vascular smooth muscle cell
US FDA	United States Food and Drug Administration
ZnPc	zinc phthalocyanine
γFe2O3	maghemite
